# Supplementary material for: The structure of plastocyanin tunes the midpoint potential by restricting axial ligation of the reduced copper ion
Source: Commun Chem. 2023 Aug 23;6:175. doi: 10.1038/s42004-023-00977-4 (PMC10447441; doi:10.1038/s42004-023-00977-4)
Supplement: Supplementary file 2 — Supplementary Information file [file 42004_2023_977_MOESM2_ESM.pdf]

Supplementary Information for

The structure of plastocyanin tunes the midpoint potential by restricting axial ligation of the reduced copper ion

Claire C. Mammoser, Brynn E. LeMasters, Sydney G. Edwards, Emma M. McRae, M. Hunter Mullins, Yiqi Wang, Nicholas M. Garcia, Katherine A. Edmonds, David P. Giedroc, and Megan C. Thielges

I. Supplementary Methods

Pc homology S2
Characterization of protein preparations S2
Assessment of Cu(II) content S2
Redox titrations S3
NMR spectroscopy S4
FT IR spectroscopy S4
Freeze-thaw treatment S6
Addition of denaturant S6
Time-dependent appearance of reduced state S7

II. Supplementary Figures and Tables

Fig. S1. Structural homology of Pc S9
Fig. S2. Sequence homology of Pc S10
Fig. S3. SDS-PAGE gel of Pc mutants S11
Fig. S4. Mass spectra S12
Fig. S5. Assessment of Cu(II) content S13
Fig. S6. Nernst plots from chemical redox titrations S14
Fig. S7. HSQC spectra of ^{15}N Pc S15
Fig. S8. FT IR spectrum of d_4-cystine S16
Table S1. Parameters from Gaussian fits to spectra of d_2Cys89 S16
Table S2. Parameters from Gaussian fits to spectra of d_3Met97 S17
Fig. S9. Correlation of E_m to linewidth of d_3Met97 S17
Fig. S10. Alternate Gaussian fits to spectra of d_3Met97 for reduced Pc S18
Table S3. Parameters from alternate Gaussian fits to spectra of d_3Met97 for reduced Pc. S19
Fig. S11. d_2Cys89 and d_3Met97 FT IR spectra following freeze-thaw S20
Fig. S12. d_2Cys89 FT IR spectra of wt Pc in denaturing buffer S20
Fig. S13. d_2Cys89 and d_3Met97 FT IR spectra of Pc over time S21
Table S4. Parameters from Gaussian fits to spectra S21
Fig. S14. Visible spectra of chemical reoxidation of Pc mutants S23

III. Supplementary References S23

I. Supplemental methods

Pc homology. Sites for mutation were selected to disrupt hydrogen bonds between and within loops of the Pc cupredoxin fold. Potential interactions disrupted are shown in Fig. S1. Asn33 and Asn34 are generally conserved, while Ser9 is not conserved in higher plants such as spinach (Fig. S2)[1].

Characterization of protein preparations. Purified samples were analyzed via SDS-PAGE (Fig. S3). Mass spectra were acquired on a Bruker Autoflex III Smartbeam MALDI-ToF spectrometer in α-cyano-4-hydroxycinnamic acid matrix (Fig. S4).

Assessment of Cu(II) content. Cu(II) content of the protein preparations was determined using 1-(2-pyridylazo)-2-naphthol (PAN) as a visible chelating indicator (Fig. S5). PAN binds Cu(II) ions in a 1:1 ratio, and Cu(II) chelation results in an increase in the visible absorption wavelength of PAN from 475 to 570 nm[2]. A stock solution of 200 μM PAN was prepared in a solution of 50% 2 mM potassium phosphate, pH 7 and 50% ethanol. This stock solution was mixed in a 1:1 ratio with solutions of $CuSO_4$ in 2 mM potassium phosphate, pH 7 and triplicate visible spectra were acquired (Cary 300, Agilent, Santa Clara, CA) to build a calibration curve with 100 μM PAN and 0-100 μM Cu(II) (Fig. S5a). Absorbances of these solutions at 553 nm varied linearly with Cu(II) concentration (Fig. S5b). The concentrations of wt and mutated Pc were determined by their absorbance at 280 nm ($\varepsilon = 4.59\ mM^{-1}cm^{-1}$). Pc was diluted to 75 μM in freshly prepared 8 M urea to denature the protein. Mutated Pc lost its blue color almost immediately upon addition of urea, while wt Pc took approx. 15 min. to become colorless. Samples were mixed in a 1:1 ratio with the 200 μM PAN stock solution, yielding final solutions with 100 μM PAN and 37.5 μM Pc in 25%

ethanol and 75% 2 mM potassium phosphate, pH 7. Visible spectra of these solutions were taken in triplicate, and the absorbance at 553 nm was used to obtain a Cu(II) concentration for each sample based on the calibration curve (Fig. S5c). wt Pc had 0.69 ± 0.04 Cu(II) ions per Pc, S9A had 0.89 ± 0.02, N34A had 0.873 ± 0.003, and N33A had 0.969 ± 0.009. We note that the lower apparent Cu(II) content in wt Pc could result from protein which is not fully denatured. We and others have observed that addition of denaturant can result in reduced protein which does not have a blue color but does retain its Cu ion. Cu in this state would not be accessible for chelation to PAN, resulting in the somewhat lower Cu(II) content observed in wt Pc. Otherwise, the results indicate approximately one Cu(II) ion present in each molecule of Pc.

Redox titrations. Titrations were performed using 5 μM Cu(II) Pc in 20 mM HEPES, 50 mM sodium chloride, pH 6.8, with 1.5 mM potassium ferricyanide. 10 μM diphenylamine sulfonic acid and 2.5 μM ruthenium hexamine were added to serve as redox mediators. 50 mM sodium ascorbate was added gradually to reduce the ferricyanide to ferrocyanide. The ratio of the two species varied the potential in the cell, in turn, affecting the oxidation state of Pc. Difference spectra using the initial oxidized solution as the reference were collected at each ascorbate addition. During the titration the potential of the solution was measured using an Ag/AgCl electrode (MTC301, Hach, Loveland, CO) filled with 3 M KCl saturated with AgCl. The visible spectra were corrected so that the baseline of each was at zero by first adjusting each spectrum to the absorbance at 795 nm, adding or subtracting its deviation across each point in the spectrum. Then, a linear function was calculated between the absorbance at 795 nm and 750 nm, which has no spectral features resulting from the protein. The value of this function at each wavelength was subtracted from the spectrum to account for any increase in the baseline as wavelength decreased, which was observed

occasionally. The fraction of Pc oxidized at each ascorbate addition was determined by the difference absorbance at 600 nm divided by the difference absorbance at the titration endpoint, when all Pc is reduced. These values were treated by the Nernst equation to yield a linear relationship with the cell potential reported by the electrode (Fig. S6). Potentials were converted to values reported vs. NHE by addition of 207 mV. Equations which fit these linear relationships have y-intercepts that report E_m directly.

NMR spectroscopy. ^{15}N,^{1}H heteronuclear single quantum coherence (HSQC) spectra were recorded at 25 °C on a Bruker Avance Neo 600 MHz spectrometer equipped with a cryogenic probe in the METACyt Biomolecular NMR Laboratory. Data were collected using Topspin 4.1.3 (Bruker), and processed and analyzed on NMRbox[3], using NMRPipe[4] and NMRFAMSparky[5], respectively. Overlaid spectra are shown in Fig. S7a. Comprehensive backbone assignments of the mutant protein could not be obtained due to challenges in expressing uniformly ^{13}C-labeled Pc. Therefore, assignments of the wt and tentative assignments of the mutant were made by comparison with previous assignments of wt Pc (Fig. S7b).[6] Several resonances in the mutant either vanished or shifted into a crowded spectral region, where an approximate shift could be estimated without knowledge of exactly which peak corresponds to the specific residue. Backbone chemical shift perturbations (Δδ) caused by the mutation were thus estimated from ^{1}H and ^{15}N chemical shifts of wt and tentative ^{1}H and ^{15}N chemical shifts of the mutant using the formula $\Delta\delta=((\Delta\delta H)^2+0.2(\Delta\delta N)^2)^{1/2}$ (Fig. 3c). Significant chemical shift changes are defined as above the threshold of 0.17 ppm between the spectra of wt and N33A Pc. Samples were analyzed by MALDI mass spectrometry after analysis to confirm that little proteolysis of Pc occurred during data collection (Fig. S4c).

FT IR Spectroscopy. Pc was exchanged into 2 mM potassium phosphate buffer, pH 7, and concentrated to ~2 mM using centrifugal filters (Amicon 0.5 mL, 3 kDa molecular weight cutoff, Sigma, St. Louis, MO). Concentration was determined by the visible absorbance at 600 nm (ε = 4.4 $mM^{-1}cm^{-1}$). Cu(I) Pc was generated by addition of 200 equivalents sodium ascorbate, or in a couple cases, 50 equivalents of dithiotreitol, resulting in loss of the blue color. Ascorbate was our preferred reductant due to concerns about displacement of the Cys89 thiol ligand by dithiothreitol. However, no evidence of displacement for the Cys89 ligand was evident in the spectra of samples reduced by dithiothreitol, as the d_2Cys89 probe absorption did not shift to the frequency characteristic of the unligated residue (Fig. S8). The spectra were identical between Cu(I) Pc samples prepared by both reagents. Data for Cu(I) Pc generated by both reductants was included in this work.

IR spectra were collected at room temperature following a 15-minute purge of the instrument with dry N_2 and were averages of either 4000 (Cu(II)) or 1500 (Cu(I)) double-sided interferograms. Interferograms were obtained on a Cary 670 FT IR (Agilent, Santa Clara, CA) with a liquid nitrogen-cooled mercury-cadmium-telluride detector. Interferograms collected for buffer and each protein sample were zero-filled by a factor of 8, apodized with a Blackman-Harris 4-term function, and Fourier transform was performed using the Mertz algorithm for phase correction, as previously described[7]. Absorption spectra were calculated from the resulting sample and reference transmission spectra. Slowly varying baseline was removed by fitting the spectral region excluding the band area to a polynomial function (MATLAB 2019b). High frequency noise was removed by applying a Fourier filter.

Each baseline-corrected spectrum was fit to a Gaussian function (Table S1, Table S2). For d_3Met97 of Cu(I) Pc the linewidth of the fit to the C-D absorption increases corresponding with the protein E_m (Fig. S9, Table S2). To assess whether two bands may underlie the broader linewidths, we fit each spectrum to a sum of two Gaussian functions (Fig. S10, Table S3). When none of the parameters (amplitude, linewidth, frequency) of the two Gaussian functions are restricted, the spectra best fit to a band with nearly the same frequency as the single Gaussian fit and a second band of 2-6% relative area. When the frequency and linewidth of one band is fixed to the absorption found for wt, the spectra best with inclusion of a second band of substantial area. However, the frequency of this band increases among mutants similarly as the single Gaussian fit, showing the same correlation with E_m as observed with the fit to a single Gaussian band.

Freeze-thaw treatment. The IR spectra of oxidized Pc that had been previously frozen in liquid nitrogen and subsequently thawed indicates appearance of a reduced population (Fig. S11). Curiously, the proteins are frozen in a purified, oxidized state in aerobic, aqueous buffer solution; the reducing agent is unclear. Mass spectrometry shows no change to indicate chemical modification (Fig. S4b).

In order to assess whether the absorbance observed results from reduced Pc rather than a denatured or apoprotein state, we collected a spectrum of 50 mM d_4-cystine in 500 mM HCl (Fig. S8). The high concentration is required due to the breadth of the absorption, which is higher than that of Cu-ligated d_2Cys89 and makes the absorbance less easily identified. 500 mM HCl is required for solubility. The spectrum shows a single absorbance at ~2244 cm^{-1} resulting from the C-D_2 asymmetric stretch.

Addition of denaturant. Appearance of the reduced state can be induced by addition of sub-denaturing concentrations of urea (Fig. S12). Samples in 2 mM potassium phosphate, pH 7 were brought to 3-5 M urea by the addition of a freshly prepared 8 M stock. Upon incubation of the protein for several hours in 3 M urea, a small (5-15%) absorption band for the C-D asymmetric stretch of d_2Cys89 characteristic of the reduced state (2210 cm^{-1}) appears as a minor species. Addition of 5M urea leads to 63% conversion to a reduced species after four hours.

Time-dependent appearance of reduced state. In addition to Pc that had been freshly prepared and that which had undergone freeze/thaw treatment, we analyzed freshly prepared protein incubated in sealed tubes at 4°C over ~40 days (Fig. S13). A band at 2210 cm^{-1} characteristic of reduced Pc appears over time; however, the behavior of the mutant proteins in this analysis was indistinguishable from wt. The integrated areas of the absorption bands indicate ~20% reduced protein after ~10 days, an additional slight increase to ~30% population after 20 days in all except S9A Pc, then no subsequent change until the final analysis (Table S4). Oxidation upon exposure to air during sample loading likely contributes to the oxidized population detected in IR analysis. Concurrently, whiteish precipitates also appear in the samples. The E_ms of all proteins determined at time of FT IR spectroscopic analysis are unchanged from freshly analyzed protein, although we note this analysis is based on the 597 nm band so could be exclusively reporting on a subpopulation of natively folded, oxidized Pc. No consistent changes can be discerned in the 1600-1700 cm^{-1} region associated with amide I vibrations (Fig. S13).

The reduction of Pc by freeze-thaw or by extended incubation at 4°C is at least partially reversible. Visible spectra of samples that show reduced population by FT IR spectroscopy are shown in black in Fig. S14. Upon addition of 1.5 mM potassium ferricyanide, along with 10 μM

diphenylamine sulfonic acid and 2.5 μM ruthenium hexamine which act as mediators of electron transfer between the protein and ferricyanide, a second visible spectrum was taken (orange), showing an increase in the 600 nm absorption indicative of Cu(II) Pc.

II. Supplemental Figures and Tables

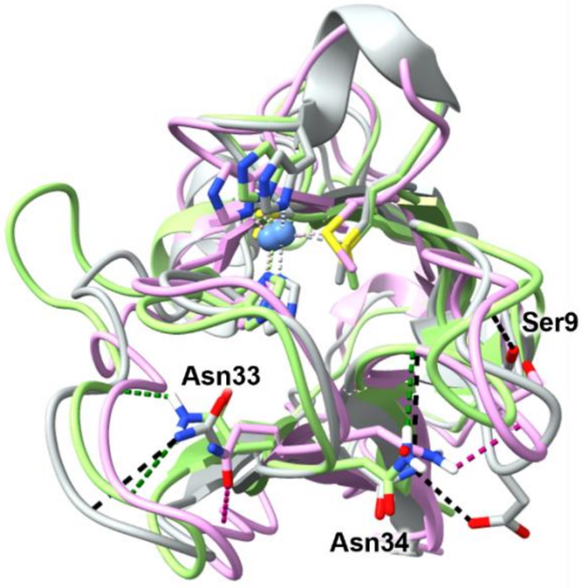

Fig. S1. Overlaid ribbon models illustrating structural homology of Pc. Grey: x-ray crystal structure (PDB: 2GIM) of Cu (II) *Nostoc* Pc. Pink: NMR structure (PDB: 1NIN) of Cu(I) *Nostoc* Pc. Green: NMR structure (PDB: 1YLB) of Cu(I) spinach Pc. Hydrogen bonding interactions of residues mutated in this study are depicted with dashed lines.

```
Ulva            ------------------------------------------------------------ 0
Arabidopsis     -MASVTSATVAIPSFTGLKASTIK-SSATVRIQTAAVASPKLTVKSS-LKNFGVAAVAAA 57
Populus         -MAAVTSAAVSIPSFTGLKAASASNAKVSASAKVSASPLPRLSIKAS-LKEVGAAVVATA 58
Phaseolus       ------------------------------------------------------------ 0
Spinacia        MATVASSAAVAVPSFTGLKASGSIKPT-TAKIIPTTTAVPRLSVKAS-LKNVGAAVVATA 58
Petroselinum    ------------------------------------------------------------ 0
Oryza           -MAALSSAAVTIPSMAPSAPGRR---------R----MRSSLVVRASLGKAAGAAAVAVA 46
Prochlorothrix  -------------------------------MKFFASLSKRFAPVLSLVV----LVAGTL 25
Phormidium      -------------------------------MKLIAQISRSLSLALFALV----LMVGSF 25
Nostoc          ------------------------------------------------------------ 0


Ulva            ----------AAIVKLGGDDGSLAFVPNNITVGAGESIEFINNAGFPHNIVFDEDAVPAG 50
Arabidopsis     ASIALAGNAMAIEVLLGGGDGSLAFIPNDFSIAKGEKIVFKNNAGYPHNVVFDEDEIPSG 117
Populus         ASAMIASNAMAVDVLLGADDGSLAFVPSEFSVPAGEKIVFKNNAGFPHNVLFDEDAVPSG 118
Phaseolus       -----------LEVLLGSGDGSLVFVPSEFSVPSGEKIVFKNNAGFPHNVVFDEDEIPAG 49
Spinacia        AAGLLAGNAMAVEVLLGGGDGSLAFLPGDFSVASGEEIVFKNNAGFPHNVVFDEDEIPSG 118
Petroselinum    -----------AEVKLGSDDGGLVFSPSSFTVAAGEKITFKNNAGFPHNIVFDEDEVPAG 49
Oryza           ASAMLAGGAMAQEVLLGANGGVLVFEPNDFTVKSGETITFKNNAGFPHNVVFDEDAVPSG 106
Prochlorothrix  LLSAAPASAATVQIKMGTDKYAPLYEPKALSISAGDTVEFVMNKVGPHNVIFDKVPA--- 82
Phormidium      VAVMSPAAAETFTVKMGADSGLLQFEPANVTVHPGDTVKWVNNKLPPHNILFDDKQVPGA 85
Nostoc          ---------ETYTVKLGSDKGLLVFEPAKLTIKPGDTVEFLNNKVPPHNVVFDAALNPAK 51
                              : :* .     : *  .::  *: : :  *   ***::**

Ulva            VDADAISA--EDYLNSKGQTVVRKLT---TPGTYGVYCDPHSGAGMKMTITVQ-  98
Arabidopsis     VDVAKISMDEQDLLNGAGETYEVALT---EPGTYSFYCAPHQGAGMVGKVTVN-  167
Populus         VDVSKISMSEEDLLNAKGETFEVALS---DKGEYTFYCSPHQGAGMVGKVIVN-  168
Phaseolus       VDAVKISMPEEELLNAPGETYVVTLD---TKGTYSFYCSPHQGAGMVGKVTVN-  99
Spinacia        VDAAKISMSEEDLLNAPGETYKVTLT---EKGTYKFYCSPHQGAGMVGKVTVN-  168
Petroselinum    VNAEKISQ--PEYLNGAGETYEVTLT---EKGTYKFYCEPHAGAGMKGEVTVN-  97
Oryza           VDVSKISQ--EEYLNAPGETFSVTLT---VPGTYGFYCEPHAGAGMVGKVTVN-  154
Prochlorothrix  -GESAPALSNTKLAIAPGSFYSVTLG---TPGTYSFYCTPHRGAGMVGTITVE-  131
Phormidium      SKELADKLSHSQLMFSPGESYEITFSSDFPAGTYTYYCAPHRGAGMVGKITVEG  139
Nostoc          SADLAKSLSHKQLLMSPGQSTSTTFPADAPAGEYTFYCEPHRGAGMVGKITVAG  105
                          .   . *.      :       * *   ** ** ****   : *
```

Fig. S2. Sequence alignment of Pc homologs. Residues of the inner coordination sphere are shown in blue. Residues mutated in the study are shown in green if matching the *Nostoc* sequence, and in red if they do not. Species included in this comparison are *Ulva prolifera* (green seaweed), *Arabidopsis thaliana*, *Populus nigra* (Lombardy poplar), *Phaseolus vulgaris* (French bean), *Spinacia oleracea* (spinach), *Petroselinum crispum* (parsley), *Oryza sativa japonica* (rice), *Prochlorothrix hollandica*, *Phormidium laminosum*, and *Nostoc*.

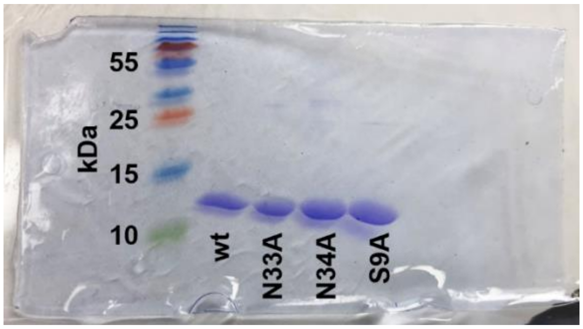

Fig. S3. SDS-PAGE analysis of Pc preparations.

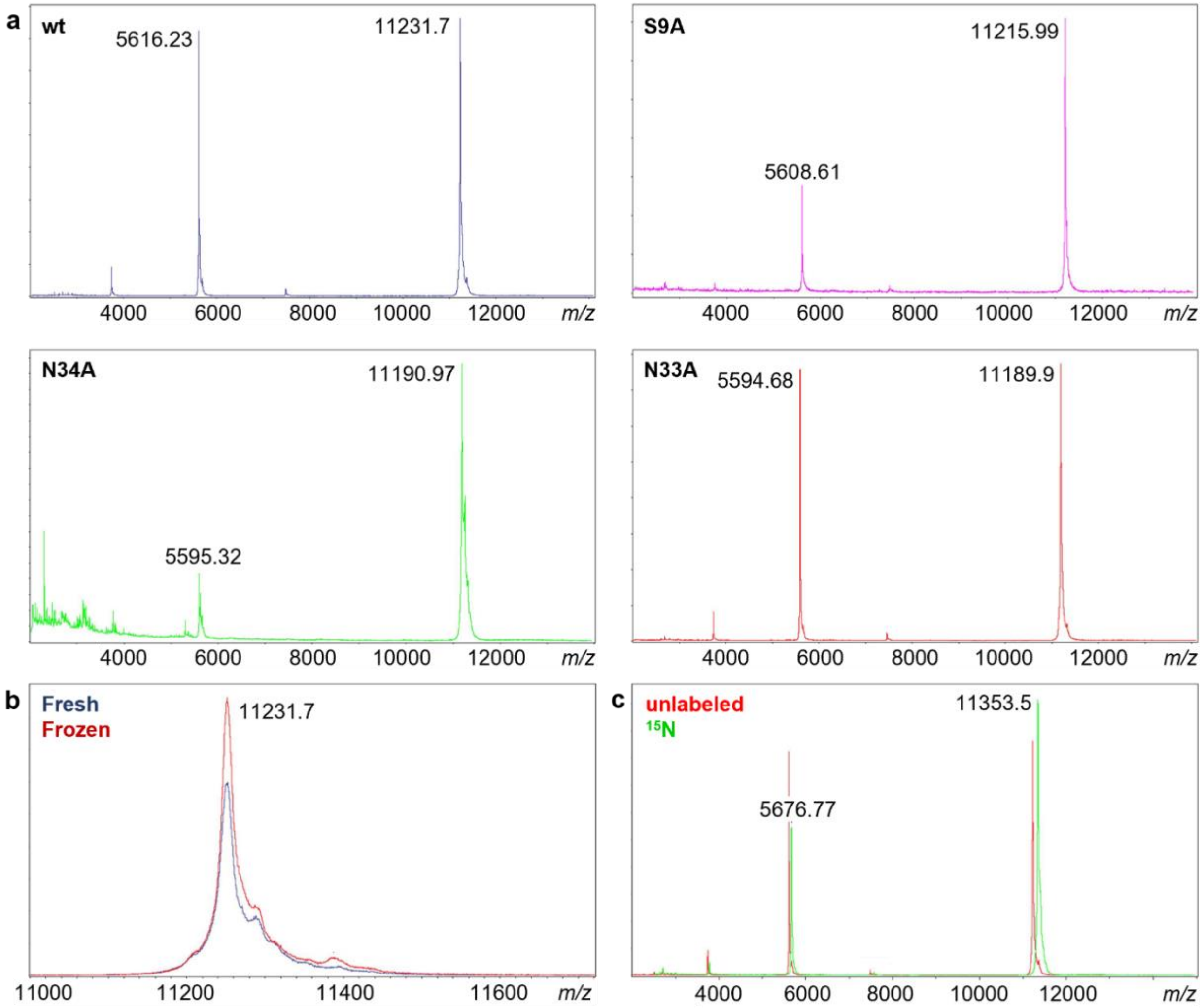

Fig. S4. MALDI mass spectra of wt and mutant Pc. (a) Observed *m/z* values reflect singly and doubly charged Pc with M67L substitution, truncation of N-terminal Met1 and Ala2 residues in expressed construct, deuteration at d_2Cys89 and d_3Met97, and inclusion of a Cu ion. Predicted average singly charged *m/z* are wt, 11225.27 Da; S9A, 11209.27 Da; N34A, 11182.25 Da; N33A, 11182.25 Da. A small deviation of ~6 Da is observed between predicted and observed singly charged *m/z,* however, the shifts in mass between Pc mutants are consistent with the mass change predicted from the mutation. Spectra in are of previously frozen protein. (b) Comparison of fresh (blue) and previously frozen (red) wt Pc. No shift in *m/z* is observed between these samples. (c) Unlabeled (red) and ^{15}N (green) Pc. Predicted average mass for ^{15}N-labeled Pc is 11350.27 Da.

Spectrum of ^{15}N-labeled Pc was obtained after collection of NMR spectra, indicating no substantial proteolysis over the course of the experiment.

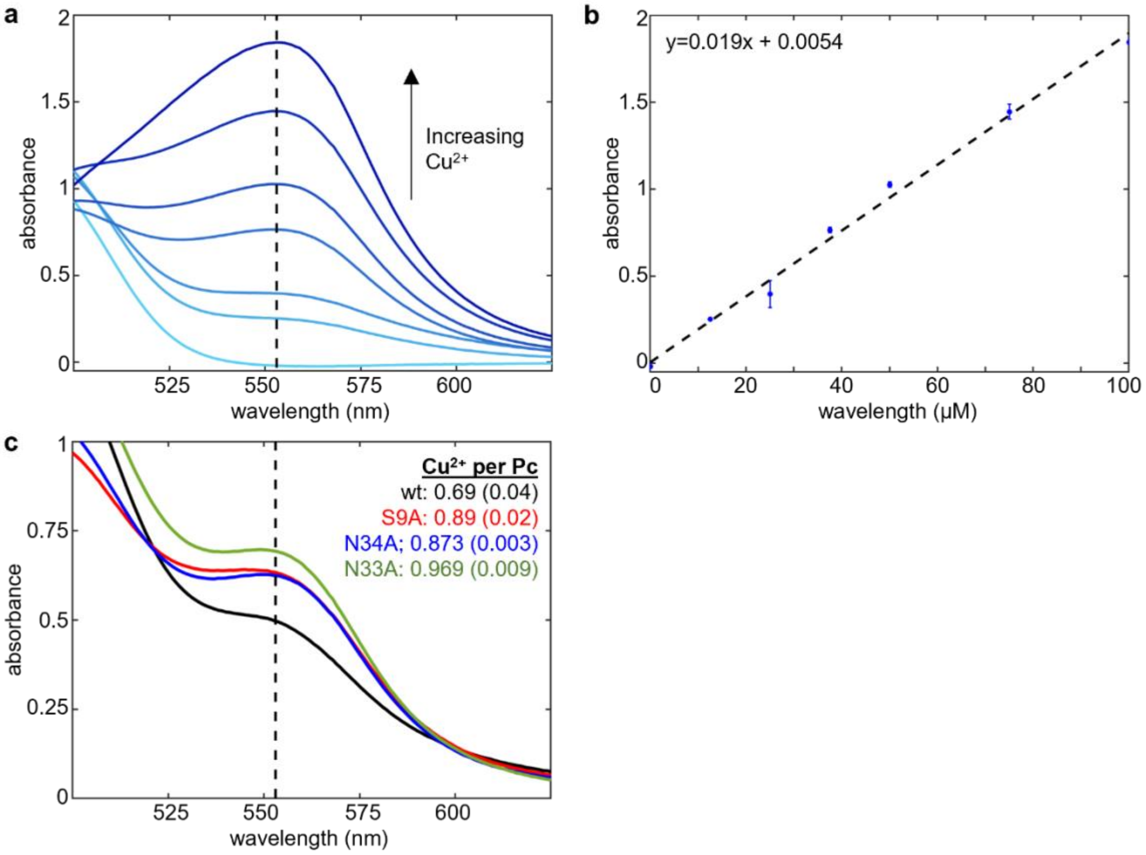

Fig. S5. Visible spectrophotometric assay of Cu(II) content in Pc. (a) Spectra of solutions of 100 µM PAN and known concentrations of Cu(II) ranging from 0-100 µM. Vertical dashed line indicates the wavelength (553 nm) from which the signal of Cu(II)-bound PAN was measured. (b) Linear fit to the absorbance at 553 nm of the Cu(II)-PAN solutions plotted against Cu(II) concentration. (c) Spectra of denatured Pc (37.5 µM based on absorbance at 280 nm) solutions with 100 µM PAN. Absorbances at 553 nm indicate a ~1:1 ratio of Pc to Cu(II) ions.

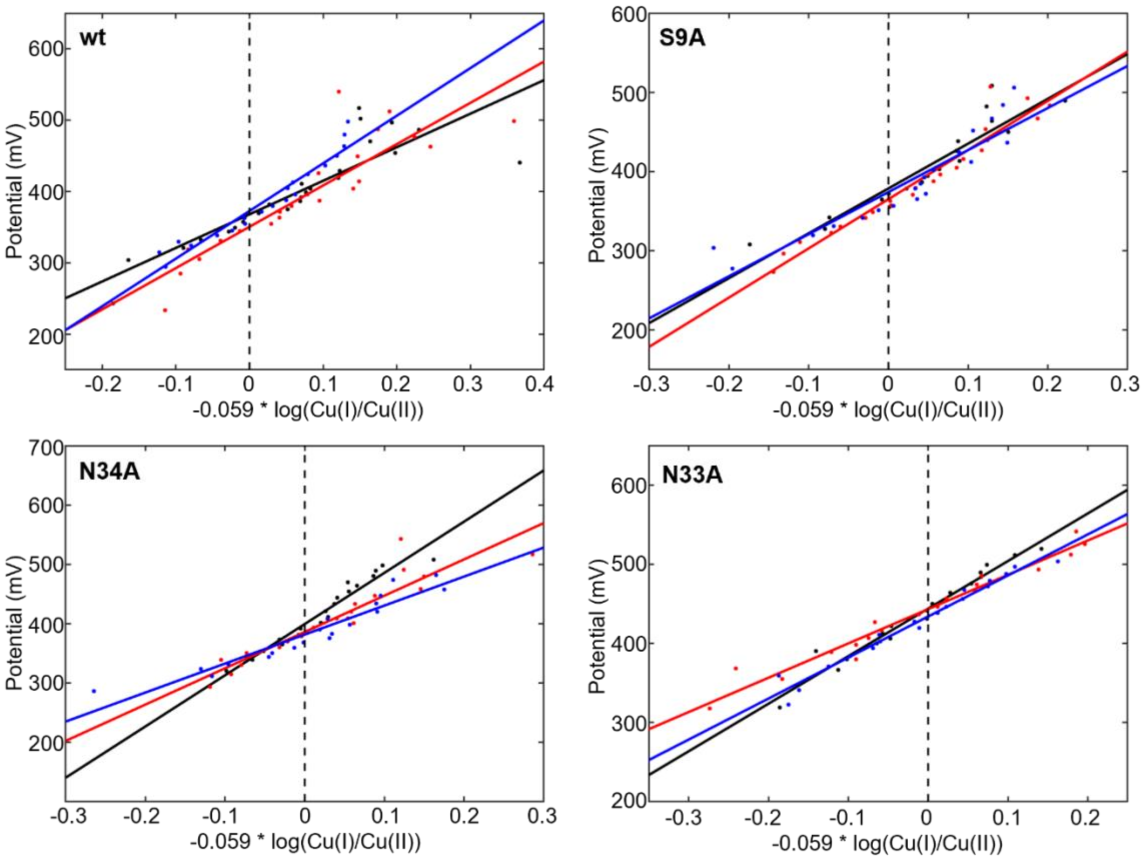

Fig. S6. Nernst plot of Cu(I):Cu(II) ratio and cell potential vs. NHE. Y-intercept (shown as dotted line) gives E_m of the redox transition. Results from three titrations are shown on each plot, with linear fits (solid lines) indicated in the same color.

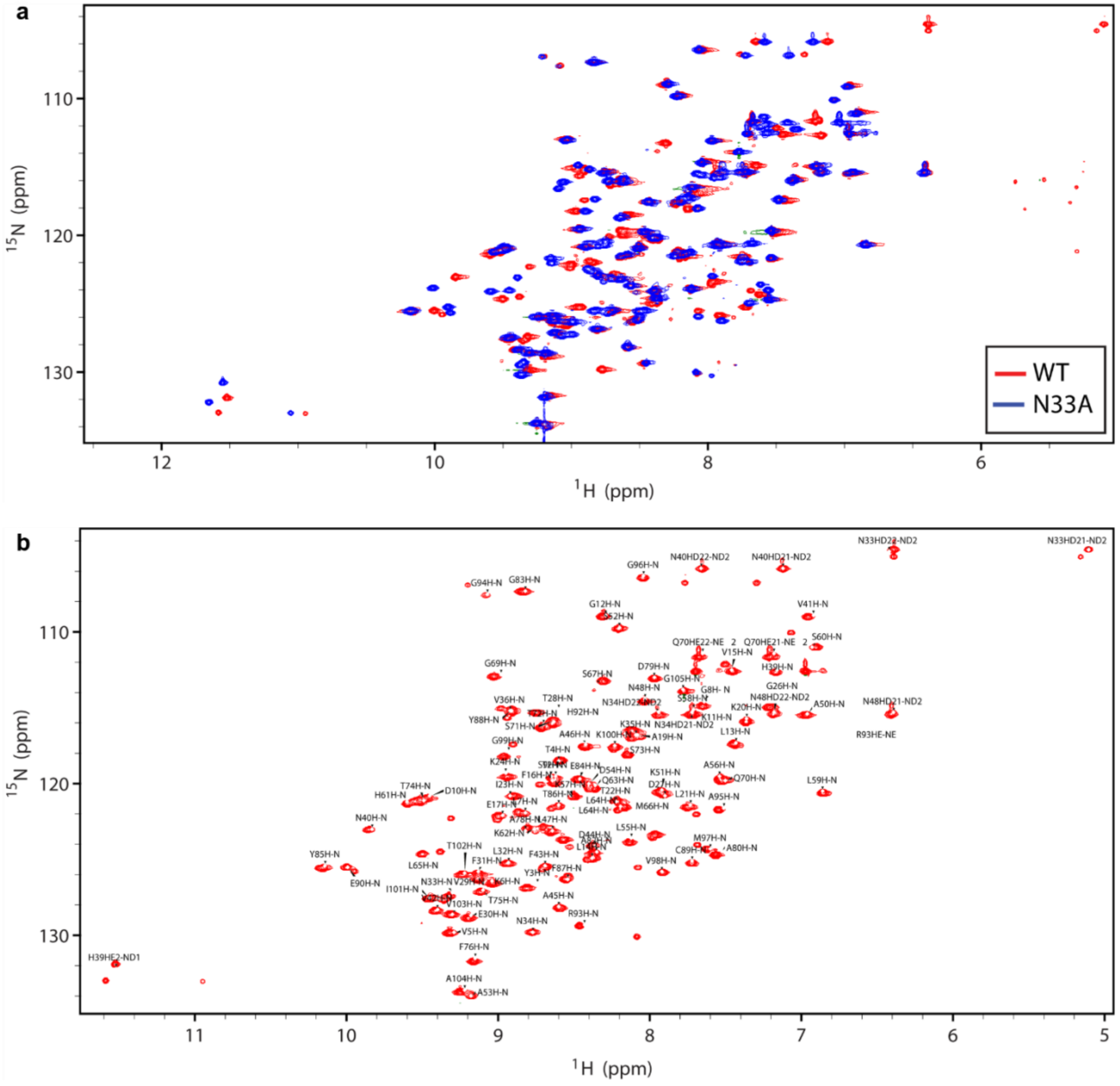

Fig. S7. ^{1}H^{15}N HSQC spectra of Pc. a) Overlaid spectra of wt (red) and N33A (blue) Pc. b) wt Pc with residue assignments.

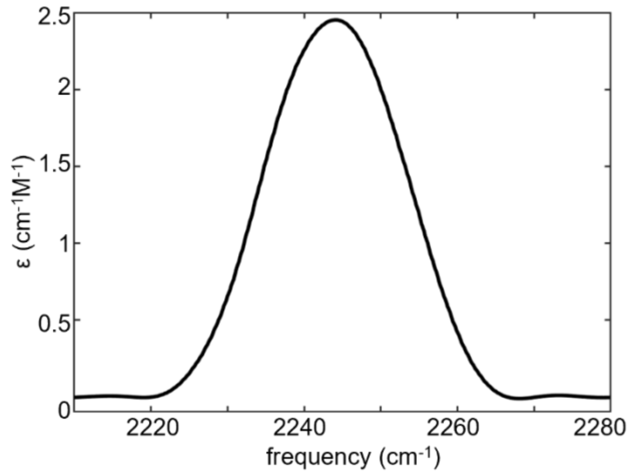

Fig. S8. Extinction spectrum of the C-D asymmetric stretch of *d*$_4$-cystine in 500 mM HCl.

Table S1. Parameters from Gaussian fits to IR spectra of *d*$_2$Cys89[a]

	d$_2$Cys89 Cu(II)		*d*$_2$Cys89 Cu(I)	
	ν_{as} (cm^{-1})	full-width-at-half-max (cm^{-1})	ν_{as} (cm^{-1})	full-width-at-half-max (cm^{-1})
wt	2235.1 (0.2)	9.1 (0.3)	2209.4 (0.3)	8.4 (0.3)
S9A	2234.9 (0.4)	9.9 (0.5)	2209.7 (0.1)	7 (1)
N34A	2235.2 (0.3)	10.43 (0.04)	2209.81 (0.08)	7.6 (0.5)
N33A	2234.7 (0.3)	10 (1)	2208.8 (0.4)	8.8 (0.4)

[a]Standard deviation of triplicate data given in parentheses

Table S2. Parameters from Gaussian fits to IR spectra of d_3Met97[a]

	d_3Met97 Cu(II)		d_3Met97 Cu(I)	
	ν_s (cm^{-1})	full-width-at-half-max (cm^{-1})	ν_s (cm^{-1})	full-width-at-half-max (cm^{-1})
wt	2126.30 (0.02)	5.40 (0.07)	2123.32 (0.05)	5.3 (0.2)
S9A	2126.30 (0.01)	5.30 (0.04)	2123.8 (0.1)	5.5 (0.3)
N34A	2126.50 (0.03)	5.48 (0.06)	2123.92 (0.08)	6.0 (0.3)
N33A	2126.23 (0.03)	5.96 (0.09)	2124.3 (0.1)	6.3 (0.5)

[a]Standard deviation of triplicate data given in parentheses

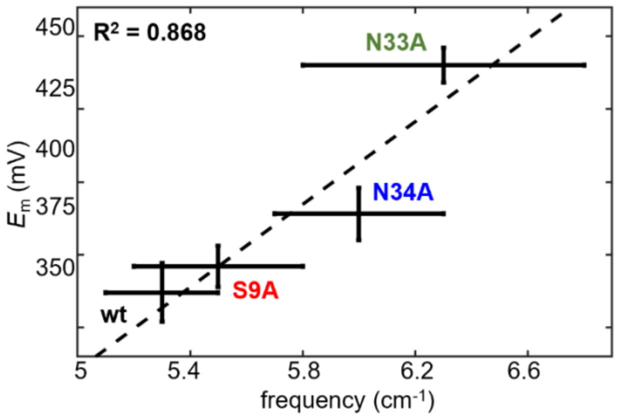

Fig. S9. Plot of E_m vs. the full-width-at-half maximum of the C-D symmetric stretch absorption of d_3Met97 for wt and mutant Pc. Error bars depict standard deviation from triplicate measurements.

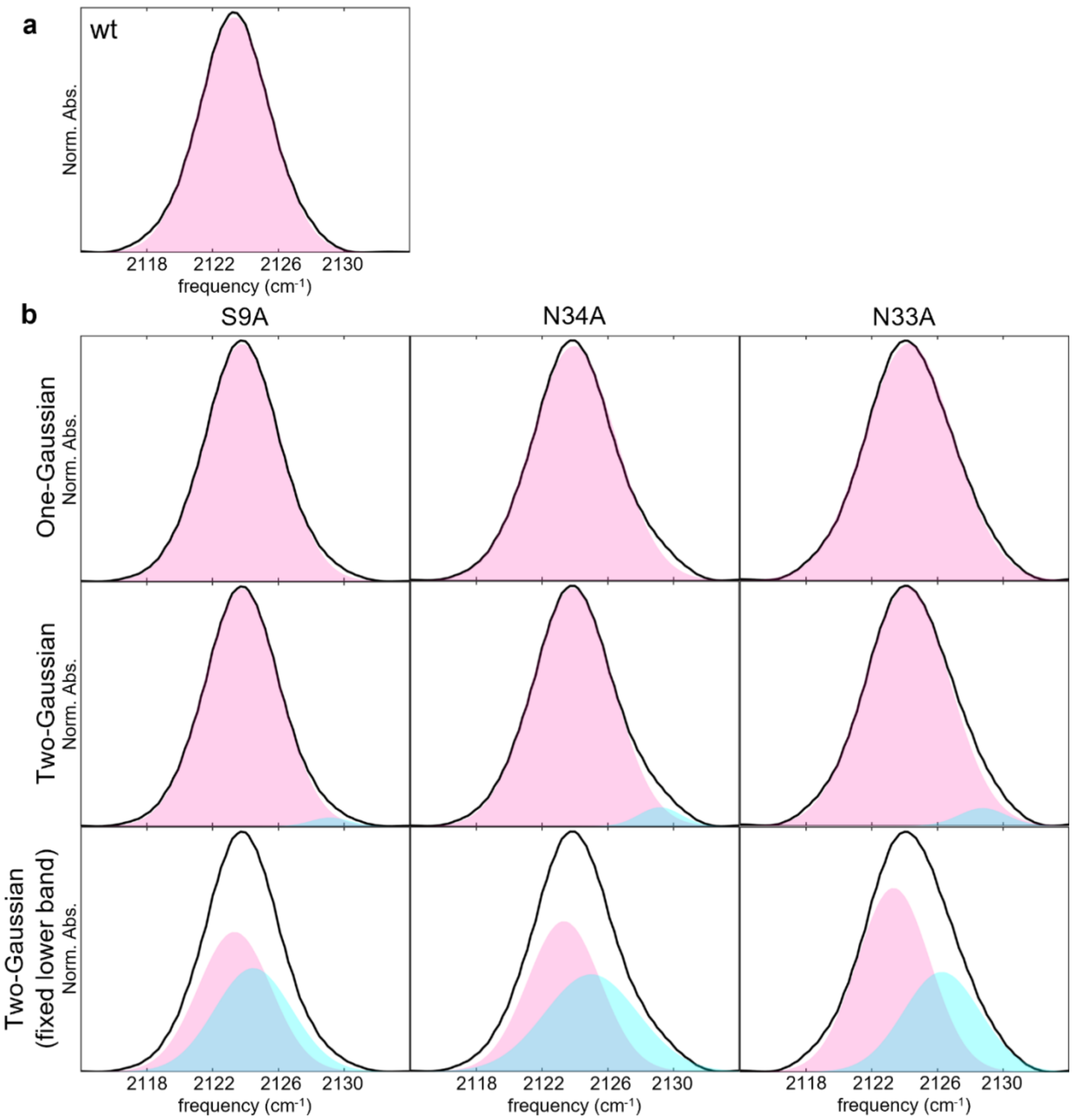

Fig. S10. Overlay of IR absorptions and component band(s) from fit to C-D symmetric stretch of *d*3Met97 for a) wt and b) mutated Cu(I) Pc. Experimental data are shown as black lines and fitted components are shown as shaded bands. Spectra were fit to one Gaussian band (top row), a sum of two Gaussian bands with no parameters fixed (middle row), or a sum of two Gaussian bands

with the frequency and linewidth of the lower frequency band fixed to that observed for wt Cu(I) Pc (bottom row).

Table S3. Parameters from Gaussian fits to IR spectra of d_3Met97 of Cu(I) Pc.[a]

	**1-Gaussian Fit**		
	Freq. (cm^{-1})		
**wt**	2123.31 (0.05)		
**S9A**	2123.8 (0.1)		
**N34A**	2123.92 (0.08)		
**N33A**	2124.3 (0.1)		
	**2-Gaussian Fit (unrestricted)**		
	Freq. 1 (cm^{-1})	Freq. 2 (cm^{-1})	% area band 2
**S9A**	2123.79 (0.08)	2129.2 (0.3)	2 (1)
**N34A**	2123.85 (0.06)	2129.14 (0.05)	3.9 (0.4)
**N33A**	2124.09 (0.02)	2128.4 (0.5)	6 (5)
	**2-Gaussian Fit (lower band fixed)**		
	Freq. 1 (cm^{-1})	Freq. 2 (cm^{-1})	% area band 2
**S9A**	2123.3	2124.3 (0.7)	64 (22)
**N34A**	2123.3	2125.0 (0.2)	42 (6)
**N33A**	2123.3	2126 (1)	46 (16)

[a]Standard deviation of triplicate data given in parentheses

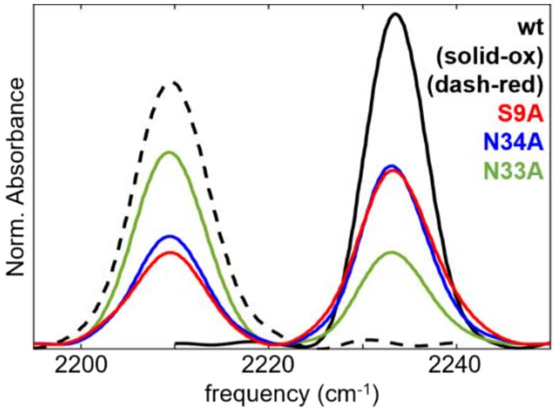

Fig. S11. Spectra showing C-D asymmetric stretch absorptions of d_2Cys89 for wt and mutant Pc following treatment by freeze-thaw.

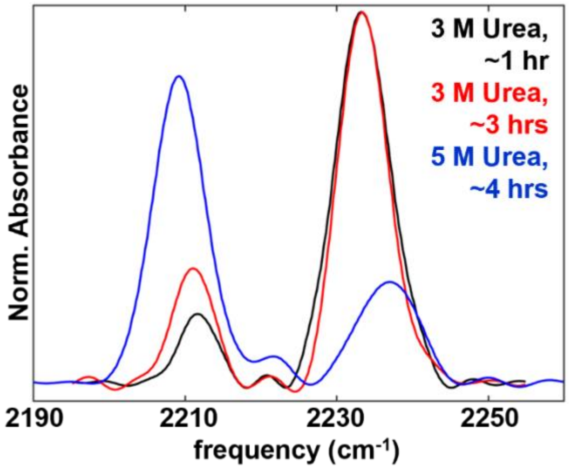

Fig. S12. IR spectra of the C-D symmetric stretch of d_2Cys89 of wt Pc upon incubation with urea.

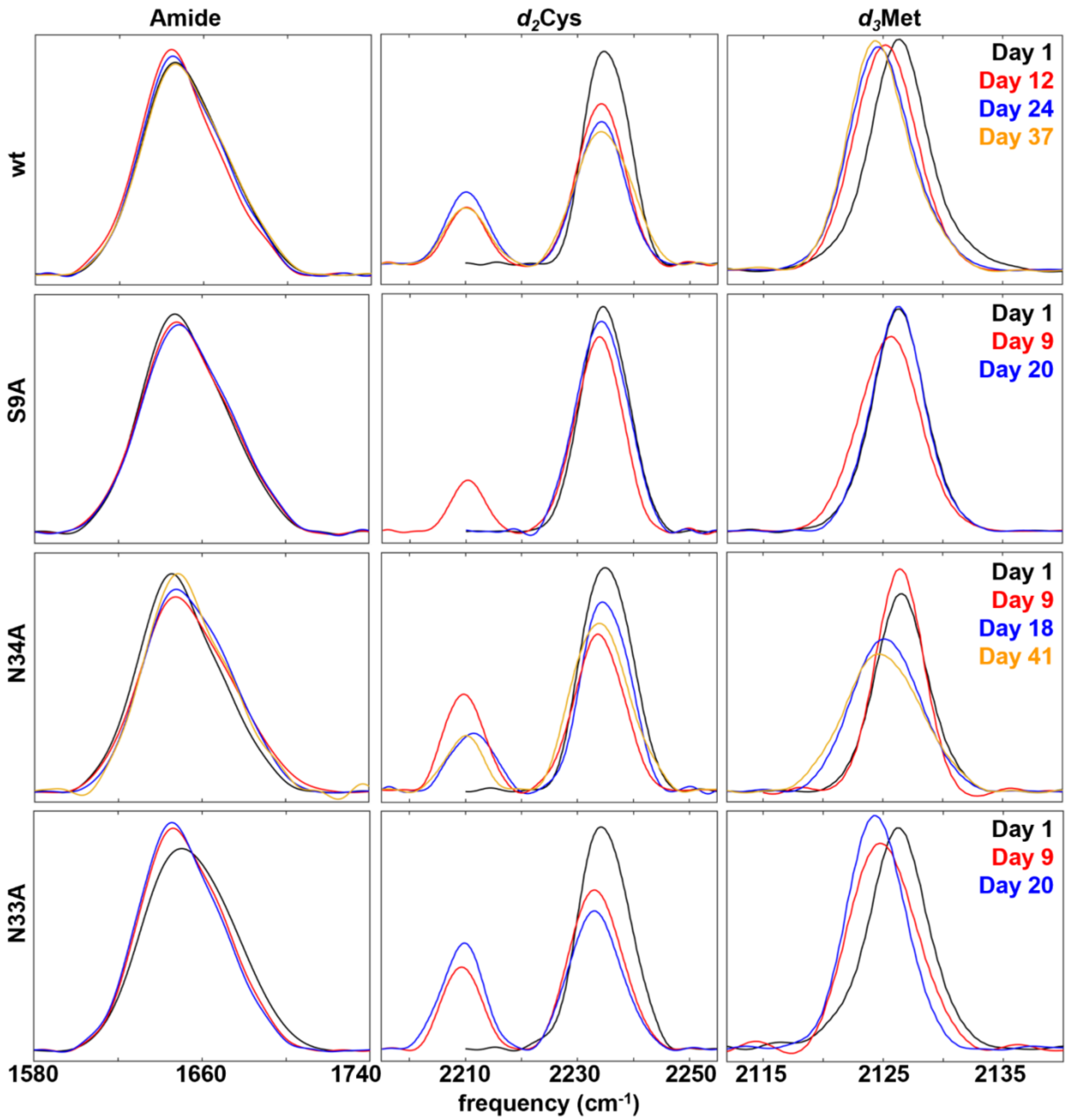

Fig. S13. IR absorptions of the amide I, C-D asymmetric stretch of d_2Cys89, and C-D symmetric stretch of d_3Met97 over time.

Table S4. Parameters from Gaussian fits to IR spectra of oxidized Pc[a]

		d_3Met97 ν (cm^{-1})	d_2Cys89 ν (cm^{-1})	d_2Cys89 % area
wt	Day 1	2126.30 (0.02)	2235.1 (0.2)	100
	Day 12	2125.3 (0.2)	2210.1 (0.3)	22 (3)

			2234.2 (0.2)	78 (3)
	Day 24	21247. (0.1)	2210.2 (0.3)	30 (2)
			2234.16 (0.06)	70 (2)
	Day 37	2124.57 (0.07)	2210.14 (0.05)	24 (3)
			2234.5 (0.8)	76 (3)
S9A	Day 1	2126.30 (0.01)	2234.9 (0.4)	100
	Day 9	2125.54 (0.04)	2210.37 (0.03)	15 (5)
			2233.8 (0.2)	85 (5)
	Day 20	2126.22 (0.09)	2234.3 (0.3)	100
N34A	Day 1	2126.50 (0.03)	2235.2 (0.3)	100
	Day 9	2126.46 (0.03)	2211.2 (0.1)	22 (4)
			22351.(0.1)	78 (4)
	Day 18	2125.3 (0.2)	2209.7 (0.3)	33 (2)
			2233.9 (0.3)	67 (2)
	Day 40	2125.0 (0.2)	2209.8 (0.4)	17 (1)
			2234.0 (0.5)	83 (1)
N33A	Day 1	2126.23 (0.03)	2234.7 (0.3)	100
	Day 9	2124.95 (0.01)	2209.3 (0.2)	28 (2)
			2233.2 (0.4)	72 (2)
	Day 20	2124.40 (0.6)	2209.5 (0.1)	38 (4)
			2233.2 (0.1)	62 (4)

[a]Standard deviation of triplicate data given in parentheses

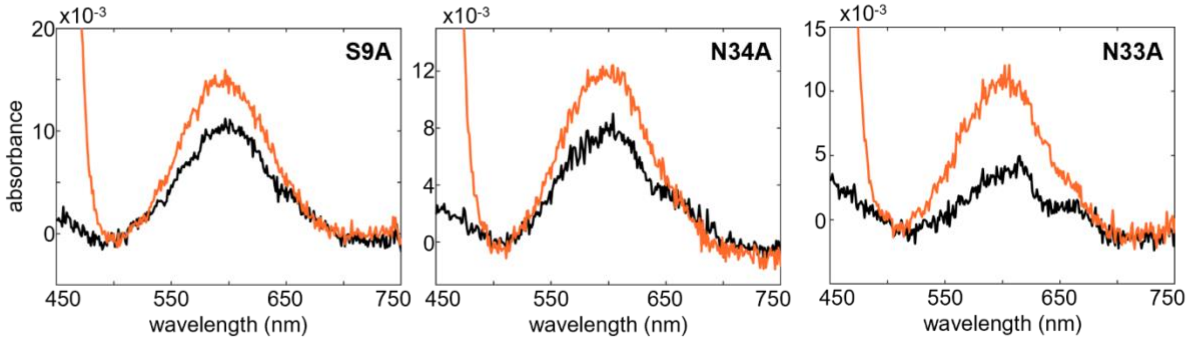

Fig. S14. Visible spectra of mutant Pc with some apparent reduced population (black) and following addition of 1.5 mM chemical oxidant potassium ferricyanide (orange) and redox

mediators as described for chemical redox titrations in 20 mM HEPES, 50 mM sodium chloride, pH 6.8. Protein is at the same concentration in both spectra. Higher intensity of the characteristic 600 nm absorption following addition of the oxidant indicates the transition to the reduced state is reversible. Asymmetry at higher wavelengths (~650 nm) is due to a known artifact associated with poor background matching in our cuvettes.

III. Supplementary References

1 Sykes, A. G. Tilden Lecture. Structure and electron-transfer reactivity of the blue copper protein plastocyanin. *Chemical Society Reviews* **14**, 283-283 (1985).

2 Anderson, R. G. & Nickless, G. Heterocyclic azo dyestuffs in analytical chemistry. A review. *The Analyst* **93**, 207-207 (1967).

3 Maciejewski, M. W. *et al.* NMRbox: A Resource for Biomolecular NMR Computation. *Biophys J* **112**, 1529–1534 (2017).

4 Delaglio, F. *et al.* NMRPipe: A multidimensional spectral processing system based on UNIX pipes. *Journal of Biomolecular NMR* **6**, 277-293 (1995).

5 Lee, W., Tonelli, M. & Markley, J. L. NMRFAM-SPARKY: enhanced software for biomolecular NMR spectroscopy. *Bioinformatics* **31**, 1325–1327 (2015).

6 Ma, L. *et al.* Backbone dynamics of reduced plastocyanin from the cyanobacterium Anabaena variabilis: regions involved in electron transfer have enhanced mobility. *Biochemistry* **42**, 320-330 (2003).

7 Horness, R. E., Basom, E. J., Mayer, J. P. & Thielges, M. C. Resolution of Site-Specific Conformational Heterogeneity in ProlineRich Molecular Recognition by Src Homology 3 Domains. *J Am Chem Soc* **138**, 1130-1133 (2016).
