# Supplementary material for: The structure of plastocyanin tunes the midpoint potential by restricting axial ligation of the reduced copper ion
Source: Commun Chem. 2023 Aug 23;6:175. doi: 10.1038/s42004-023-00977-4 (PMC10447441; doi:10.1038/s42004-023-00977-4)
Supplement: Supplementary file 3 — Description of Additional Supplementary Files [file 42004_2023_977_MOESM3_ESM.pdf]

Description of Additional Supplementary Files

File name: Supplementary Data 1

Description: Raw data associated with chemical redox titrations

File name: Supplementary Data 2

Description: NMR spectra

File name: Supplementary Data 3

Description: FTIR data
