# Supplementary material for: The structure of plastocyanin tunes the midpoint potential by restricting axial ligation of the reduced copper ion
Source: Commun Chem. 2023 Aug 23;6:175. doi: 10.1038/s42004-023-00977-4 (PMC10447441; doi:10.1038/s42004-023-00977-4)
Supplement: Supplementary file 5 — Supplementary Data 2 [file 42004_2023_977_MOESM5_ESM.pdf]

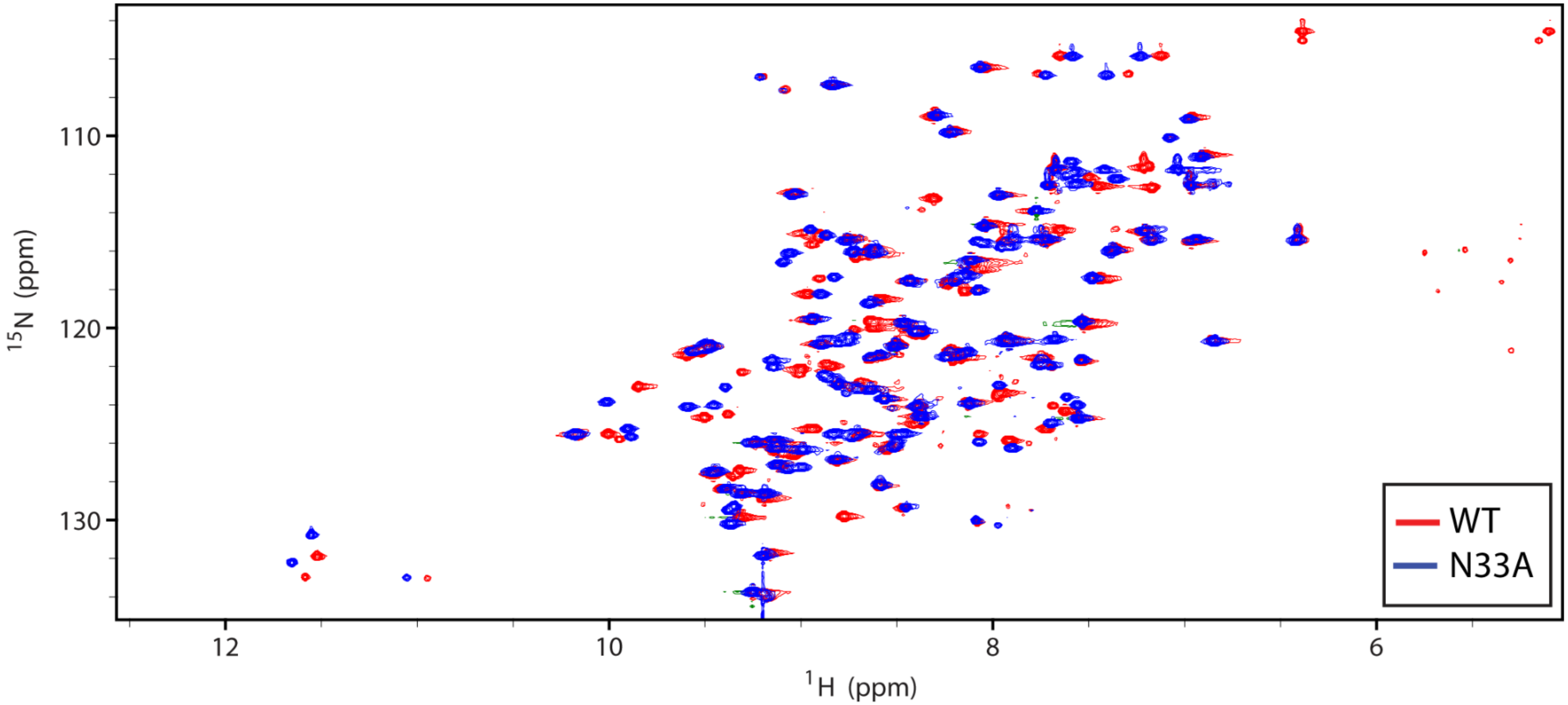

Information regarding sample preparation and data acquisition are provided in methods and supporting information.

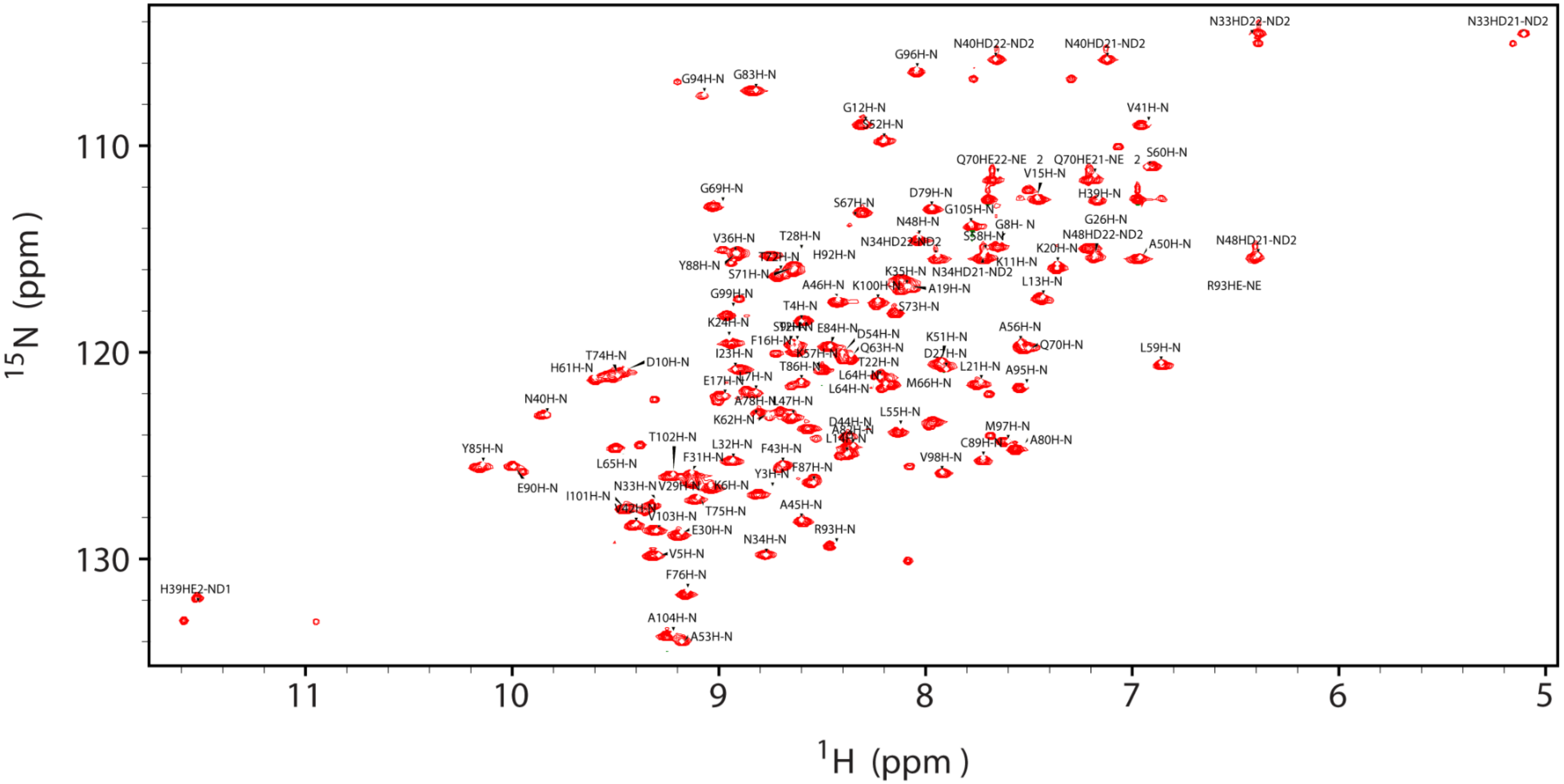

Spectrum is of wt Pc with resonances assignments indicated.
