# Supplementary material for: The structure of plastocyanin tunes the midpoint potential by restricting axial ligation of the reduced copper ion
Source: Commun Chem. 2023 Aug 23;6:175. doi: 10.1038/s42004-023-00977-4 (PMC10447441; doi:10.1038/s42004-023-00977-4)
Supplement: Supplementary file 7 — Reporting Summary [file 42004_2023_977_MOESM7_ESM.pdf]

nature portfolio

Corresponding author(s): Megan Thielges

Last updated by author(s): Jul 29, 2023

Reporting Summary

Nature Portfolio wishes to improve the reproducibility of the work that we publish. This form provides structure for consistency and transparency in reporting. For further information on Nature Portfolio policies, see our Editorial Policies and the Editorial Policy Checklist.

Statistics

For all statistical analyses, confirm that the following items are present in the figure legend, table legend, main text, or Methods section.

n/a	Confirmed	
☐	☒	The exact sample size (n) for each experimental group/condition, given as a discrete number and unit of measurement
☐	☒	A statement on whether measurements were taken from distinct samples or whether the same sample was measured repeatedly
☒	☐	The statistical test(s) used AND whether they are one- or two-sided<br>*Only common tests should be described solely by name; describe more complex techniques in the Methods section.*
☒	☐	A description of all covariates tested
☒	☐	A description of any assumptions or corrections, such as tests of normality and adjustment for multiple comparisons
☐	☒	A full description of the statistical parameters including central tendency (e.g. means) or other basic estimates (e.g. regression coefficient) AND variation (e.g. standard deviation) or associated estimates of uncertainty (e.g. confidence intervals)
☒	☐	For null hypothesis testing, the test statistic (e.g. F, t, r) with confidence intervals, effect sizes, degrees of freedom and P value noted<br>*Give P values as exact values whenever suitable.*
☒	☐	For Bayesian analysis, information on the choice of priors and Markov chain Monte Carlo settings
☒	☐	For hierarchical and complex designs, identification of the appropriate level for tests and full reporting of outcomes
☒	☐	Estimates of effect sizes (e.g. Cohen's d, Pearson's r), indicating how they were calculated

Our web collection on statistics for biologists contains articles on many of the points above.

Software and code

Policy information about availability of computer code

Data collection	Data collection was performed using Agilent Cary Win-UV software for all UV-Visible spectroscopy and with Agilent Resolutions Pro software for FTIR spectroscopy. NMR data was collected using Bruker Topspin 4.1.3.
Data analysis	Data analysis for UV-Visible and FTIR spectroscopic data was performed using MATLAB 2019b (Mathworks). NMR data was processed using NMRbox, using NMRPipe and NMRFAMSparky.

For manuscripts utilizing custom algorithms or software that are central to the research but not yet described in published literature, software must be made available to editors and reviewers. We strongly encourage code deposition in a community repository (e.g. GitHub). See the Nature Portfolio guidelines for submitting code & software for further information.

Data

Policy information about availability of data

All manuscripts must include a data availability statement. This statement should provide the following information, where applicable:

- Accession codes, unique identifiers, or web links for publicly available datasets
- A description of any restrictions on data availability
- For clinical datasets or third party data, please ensure that the statement adheres to our policy

The protein structure used in this study was obtained from the RCSB Protein Data Bank (2GIM). FTIR data used in this study are available as Supplementary Data File

1. Raw data associated with chemical redox titrations are available as Supplementary Data File 2. NMR spectra are available in Supplemental Data File 3. Data supporting this study are also available by request to the corresponding author.

Research involving human participants, their data, or biological material

Policy information about studies with human participants or human data. See also policy information about sex, gender (identity/presentation), and sexual orientation and race, ethnicity and racism.

Reporting on sex and gender	No human participants were involved in the study.
Reporting on race, ethnicity, or other socially relevant groupings	No human participants were involved in the study.
Population characteristics	No human participants were involved in the study.
Recruitment	No human participants were involved in the study.
Ethics oversight	No human participants were involved in the study.

Note that full information on the approval of the study protocol must also be provided in the manuscript.

Field-specific reporting

Please select the one below that is the best fit for your research. If you are not sure, read the appropriate sections before making your selection.

☒ Life sciences ☐ Behavioural & social sciences ☐ Ecological, evolutionary & environmental sciences

For a reference copy of the document with all sections, see nature.com/documents/nr-reporting-summary-flat.pdf

Life sciences study design

All studies must disclose on these points even when the disclosure is negative.

Sample size	Triplicate samples were independently measures for all carbon-deuterium FTIR spectra, and duplicate samples were independently measured for amide I data. Chemical redox titrations were performed in triplicate.
Data exclusions	All relevant data obtained was included in the analysis.
Replication	All data were obtained in replicate as described above and in the text. Data obtained on equivalent samples was highly reproducible.
Randomization	Randomization is not relevant to our study as all samples are recombinantly expressed in our laboratory from the same source genetic material.
Blinding	Blinding is not relevant to our study because our conclusions are drawn from spectroscopic data which are fit using standard methods across all samples.

Reporting for specific materials, systems and methods

We require information from authors about some types of materials, experimental systems and methods used in many studies. Here, indicate whether each material, system or method listed is relevant to your study. If you are not sure if a list item applies to your research, read the appropriate section before selecting a response.

Materials & experimental systems

n/a	Involved in the study	
☒	☐	Antibodies
☒	☐	Eukaryotic cell lines
☒	☐	Palaeontology and archaeology
☒	☐	Animals and other organisms
☒	☐	Clinical data
☒	☐	Dual use research of concern
☒	☐	Plants

Methods

n/a	Involved in the study	
☒	☐	ChIP-seq
☒	☐	Flow cytometry
☒	☐	MRI-based neuroimaging
